# Early onset esophageal adenocarcinoma: a distinct molecular entity?

**DOI:** 10.18632/oncoscience.290

**Published:** 2016-02-01

**Authors:** Anna M.J. van Nistelrooij, Ronald van Marion, PALGA-group, Katharina Biermann, Manon C.W. Spaander, J. Jan B. van Lanschot, Bas P.L. Wijnhoven, Winand N.M. Dinjens

**Affiliations:** ^1^ Department of Pathology, Erasmus MC Cancer Institute, University Medical Center Rotterdam, The Netherlands; ^2^ Department of Surgery, Erasmus MC Cancer Institute, University Medical Center Rotterdam, The Netherlands; ^3^ Members of the PALGA-group: I. van Lijnschoten, Pathology and Medical Microbiology, PAMM Institute, Eindhoven, The Netherlands. M. Hogenes, Laboratory of Pathology, Oost-Nederland, Hengelo, The Netherlands; ^4^ Department of Gastroenterology and Hepatology, Erasmus MC Cancer Institute, University Medical Center Rotterdam, The Netherlands

**Keywords:** esophageal adenocarcinoma, early onset cancer, molecular analysis

## Abstract

Esophageal adenocarcinoma (EAC) is typically diagnosed in elderly with a median age of 68 years. The incidence of EAC has been rising over the last decades, also among young adults. The aim of the study was to investigate whether early onset EAC is a distinct molecular entity.

To identify early onset EACs, the nationwide network and registry of histo- and cytopathology in the Netherlands (PALGA) was searched. Twenty-eight tumors of patients aged ≤40 years were selected and matched with 27 tumors of patients aged ≥68 years. DNA was isolated from surgically resected specimen and sequenced on the Ion Torrent Personal Genome Machine with the Ion AmpliSeq Cancer Panel.

No differences in mutational load between early onset and conventional EACs were observed (*P*=0.196). The most frequently mutated genes were *TP53* (73%) and *P16* (16%). Additional mutations in early onset EACs occurred exclusively in: *APC*, *CDH1, CTNNB1*, *FGFR2*, and *STK11*. In the conventional EACs additional mutations were exclusively identified in: *ABL1*, *FBXW7*, *GNA11*, *GNAS*, *KRAS*, *MET*, *SMAD4*, and *VHL*.

Additional mutations besides *TP53* and *P16* seem to occur in different genes related to cell fate pathways for early onset EACs, while the additional mutations in conventional EACs are related to survival pathways.

## INTRODUCTION

Esophageal adenocarcinoma (EAC) is typically diagnosed in elderly adults with a median age of 68 years [1]. The incidence of EAC has been rising rapidly over the last decades, also among young adults [2, 3]. The clinicopathological characteristics of these young adults with early onset EACs are different compared to the conventional EAC of the older patients: those with early onset EAC suffer from less co-morbidities, present with advanced disease stages more often, undergo more aggressive treatments, but ultimately obtain the same relative five-year survival rates as their older counterparts [4-6]. Individuals can be predisposed to early onset EAC through heredity, since seven percent of the patients diagnosed with EAC can be considered Familial Barrett's Esophagus (FBE), of which the age at diagnosis is generally lower compared to sporadic cases [7, 8], yet no genetic defects related to FBE have been identified. Nevertheless, early onset EAC can be sporadic as well, lacking a genetic predisposition.

It has been accepted that cancer is generally a disease of the elderly population. In addition, evidence is obtained that the transformation of a normal cell into a malignant cell and subsequently the outgrowth to a clinically manifest lesion takes several decades [9]. This transformation process is driven by genomic instability leading to the accumulation of mutations. About three mutations in driver genes, which are causally involved in the tumorigenic process, have to accumulate to induce this malignant transformation [10]. As a result of the genomic instability also passenger mutations, which are not involved in the tumorigenic process, will accumulate. Hence, it can be anticipated that early onset EACs went through an accelerated transformation process and as a result could have a lower mutational load of (passenger) mutations, as has been reported for other tumors before [11]. In addition, it is possible that early onset EAC is a distinct molecular entity as has been demonstrated e.g. for colorectal carcinoma [12].

The aim of the present study was to investigate whether early onset EAC is a distinct molecular entity. By next-generation sequencing with a standard cancer panel the mutational load and molecular profile of early onset EACs was determined and compared with the conventional EACs.

## RESULTS

Thirty-seven patients diagnosed with EAC or adenocarcinoma of the gastro-esophageal junction (GEJ) and aged ≤40 years at time of diagnosis were identified in the PALGA database. Twenty-eight samples obtained from these patients passed quality controls and were included in the study (mean age: 37.2 years, range 28-40 years, 89% male). Twenty-seven patients diagnosed with EAC or adenocarcinoma of the GEJ and aged ≥68 years at time of diagnosis were matched with patients aged ≤40 years based on TNM-stage and tumor differentiation grade (Mean age: 74.6 years, range 68-83 years, 78% male). All tumors were tested microsatellite stable. Of the patients aged ≤40 years seven received some form of neoadjuvant therapy, while none of the patients aged ≥68 years did. Patients- and tumor characteristics are listed in Table 1 according to age groups.

**Table 1:** Patient- and tumor characteristics according to age groups

	Early onset EAC *n* = 28 (%)	Conventional EAC *n* = 27 (%)	*p*-value (χ2)
**Mean Age (sd)**	37.21(3.023)	74.63 (4.395)	**<0.001 (*T*-test)**
**Gender**			**0.249**
Male	25 (89.3)	21 (77.8)	
Female	3 (10.7)	6 (22.2)	
**Tumor type**			**0.718**
EAC	16 (57.1)	17 (63.0)	
GEJAC	9 (32.1)	9 (33.3)	
Cardia	2 (7.1)	1 (3.6)	
Unknown	1 (3.7)	0 (0)	
TNM stage*			**0.350**
IA	5 (17.9)	3 (11.1)	
IB	3 (10.7)	0 (0)	
IIA	0 (0)	1 (3.7)	
IIB	2 (7.1)	7 (25.9)	
IIIA	9 (32.1)	7 (25.9)	
IIIB	3 (10.7)	2 (7.4)	
IIIC	5 (17.9)	6 (22.2)	
IV	1 (3.6)	1 (3.7)	
**Differentiation grade**			**0.233**
High grade dysplasia	0 (3.6)	0 (0)	
Good	4 (14.3)	0 (0)	
Moderate	11 (39.3)	16 (59.3)	
Poor	9 (32.1)	9 (33.3)	
Unknown	4 (14.3)	2 (7.4)	

EAC= esophageal adenocarcinoma

GEJAC= gastro-esophageal junction adenocarcinoma

* According to the classification of the American Joint Committee on Cancer (AJCC) Staging Manual 7^th^ edition.

Next-Generation sequencing with the Ion Torrent Personal Genome Machine (PGM) revealed 83 mutations in 55 EAC samples before filtering: 36 mutations in the patients aged ≤40 years and 47 mutations in the patients aged ≥68 years (*P* = 0.094). After filtering 78 mutations remained: 35 mutations were identified in the patients aged ≤40 years and 43 mutations in the patients aged ≥68 years. The mean number of mutations for the young adults and the older patients was, 1.25 (SD 0.844) and 1.59 (SD 1.083) respectively, and not significantly different (*P* = 0.196).

**Figure 1:**
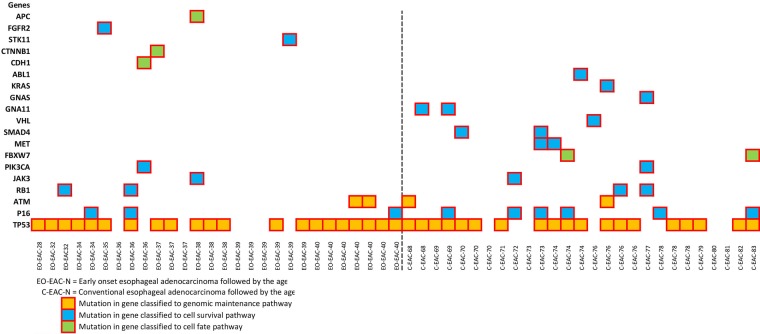
Mutations identified in early onset EACs and conventional EACs classified to cell signaling pathways

The most frequently mutated genes were *TP53* (73%), *P16* (16%), *ATM* (7%), and *RB1* (7%). In the early onset EACs *TP53* was altered in 75% and *P16* in 11%, whereas in the conventional EACs a mutation in *TP53* was found in 70% and *P16* was mutated in 22%. Except for one, all *P16* mutations occurred simultaneously with a *TP53* mutation. In 43% of the early onset EACs and in 33% of the conventional EACs no additional mutations besides a *TP53* mutations or a *P16* mutations were identified. The genes *ATM*, *JAK3*, *PIK3CA*, and *RB1* were mutated equally between both groups. Additional mutations in five individual early onset EACs occurred exclusively in the genes: *APC*, *CDH1*, *CTNNB1*, *FGFR2*, and *STK11*. In the conventional EACs additional mutations were exclusively identified in the genes: *ABL1*, *FBXW7*, *GNA11*, *GNAS*, *KRAS*, *MET*, *SMAD4*, and *VHL* (Figure 1 and Table 2).

**Table 2:** Extensive information of classification of genes in signaling pathways and cellular processes according to age groups

Gene	Gene Name	Early onset/Conventional	Classification	Corepathway	Cellular process
*ABL1*	C-abl oncogene 1, non-receptor tyrosine kinase	Conventional	Oncogene	Cell Cycle/Apoptosis	Cell Survival
*APC*	Adenomatous polyposis coli	Early onset	Tumor suppressor gene	APC	Cell Fate
*ATM*	Ataxia telangiectasia mutated	Early onset/Conventional	Tumor suppressor gene	DNADamageControl	Genome Maintenance
*CDH1*	Cadherin 1, type 1, E-cadherin (epithelial)	Early onset	Tumor suppressor gene	APC	Cell Fate
*CTNNB1*	Catenin (cadherin-associated protein), beta 1, 88kDa	Early onset	Oncogene	APC	Cell Fate
*FBXW7*	F-box and WD repeat domain containing7	Conventional	Tumor suppressor gene	NOTCH	Cell Fate
*FGFR2*	Fibroblast growth factor receptor 2	Early onset	Oncogene	PI3K; RAS; STAT	Cell Survival
*GNA11*	Guanine nucleotide binding protein (G protein), alpha 11 (Gq class)	Conventional	Oncogene	PI3K; RAS; MAPK	Cell Survival
*GNAS*	GNAS complex locus	Conventional	Oncogene	APC; PI3K; TGF-β, RAS	Cell Survival/Cell Fate
*JAK3*	Janus kinase 3	Early onset/Conventional	Oncogene	STAT	Cell Survival
*KRAS*	v-Ki-ras2 Kirsten rat sarcoma viral oncogene homolog	Conventional	Oncogene	RAS	Cell Survival
*MET*	Met proto-oncogene (hepatocyte growth factor receptor)	Conventional	Oncogene	PI3K; RAS	Cell Survival
*P16*	Cyclin-dependent kinase inhibitor 2A	Early onset/Conventional	Tumor suppressor gene	Cell Cycle/Apoptosis	Cell Survival
*PIK3CA*	Phosphatidylinositol-4,5-Bisphosphate 3-Kinase, catalytic subunit alpha	Early onset/Conventional	Oncogene	PI3K	Cell Survival
*RB1*	Retinoblastoma 1	Early onset/Conventional	Tumor suppressor gene	Cell Cycle/Apoptosis	Cell Survival
*SMAD4*	SMAD family member 4	Conventional	Tumor suppressor gene	TGF-β	Cell Survival
*STK11*	Serine/threonine kinase 11	Early onset	Tumor suppressor gene	mTOR	Cell Survival
*TP53*	Tumor protein p53	Early onset/Conventional	Tumor suppressor gene	Cell Cycle/Apoptosis; DNADamageControl	Genome Maintenance
*VHL*	Von Hippel-Lindau tumor suppressor	Conventional	Tumor suppressor gene	PI3K; RAS; STAT	Cell Survival

Adapted from Vogelstein et al. 2013 [9]

## DISCUSSION

For the first time a molecular analysis was performed on an exclusive group of patients with early onset EACs, to determine whether this is a distinct entity based on molecular spectrum. In comparison with the conventional EACs no difference in the total mutational load, including common driver mutations, was observed in the early onset EACs. Although no evidently differences were observed between the two groups with regard to molecular profile, the additional mutations, besides mutations in *TP53* and *P16*, identified in some individual early onset EACs differed when compared to the additional mutations identified in the conventional EACs.

Presently, there is no accepted clear definition of early onset EACs. Recent publications demonstrate that 5% of the patients diagnosed with EAC are aged ≤40 years [5] and 10% aged ≤ 50 years [6]. In these studies young adults with EAC were compared with the conventional EAC patients, with a median age of 68 years, based on clinicopathological characteristics, showing that these younger patients present with more advanced disease stages, receive more often aggressive treatment regimes, however, ultimately obtain relative survival rates comparable with their older counterparts [6]. In order to ensure a clear segregation of the two entities and to avoid any overlap, a more restrictive definition of both early onset EAC and conventional EAC was used and patients between 41 and 67 years were excluded in the present study.

EAC evolves from the premalignant condition Barrett's esophagus, following a multimorphological pathway, in which metaplasia evolves to low-grade dysplasia, high-grade dysplasia and ultimately into invasive adenocarcinoma, during this malignant transformation mutations accumulate over time [9, 13]. The number of mutations in a tumor originating from self-renewing tissue, e.g. the esophagus, is directly correlated with age. The majority of these mutations are passenger mutations that have no effect on the neoplastic progression. Whereas, the minority are the driver mutations, which confer a selective growth advantage. The passenger mutations occur mostly during the pre-neoplastic phase, which is evidently longer for older patients than for the younger ones [14]. Based on this concept it can be hypothesized that the number of total mutations, i.e. mutational load, is lower in early onset EACs as compared to conventional EACs.

Although it did not reached the level of significance, probably due to the relatively small amount of patients, a higher amount of total mutations (i.e. passenger and driver mutations) was observed in the conventional EACs when compared to the early onset EACs, which is in line with concept as has been described previously. The current data did not revealed a significant difference in the load of driver mutations between the two age groups. Since the use of the Cancer Hotspot panel in this study, by which only 207 gene “hot spot” regions are investigated that are frequently mutated in human cancers, not all genes were covered. Hence, a complete overview of the total mutation spectrum per patient could not be established. An alternative explanation for the comparable load of mutations between the early onset EACs and the conventional EACs can be the occurrence of an ultramutator phenotype in the young adults resulting in an accelerated accumulation of mutations. By this phenomenon, young adults with early onset EAC could bear a comparable mutational load as compared to their older counterparts despite their shorter time of tumorigenesis [15].

At a first glance no evidently differences were observed between the early onset EACs and the conventional EACs, with regard to the molecular profiles: the tumor suppressor gene *TP53* was altered in approximately 72% of EACs (75% in early onset *vs*. 70% in conventional EACs), which is comparable with other studies [16]. Mutations of *TP53* have been suggested to be an early genetic event in the multimorphological pathway of esophageal adenocarcinogenesis and are facilitating the accumulation of mutations [9, 17]. Alterations of tumor suppressor gene *P16* are additional early events in EAC, and present in approximately 12% [16]. Here, in 11% of the early onset EACs a *P16* mutation was identified, whereas in the conventional EACs a *P16* mutation was identified in 22%. A remarkable observation was made regarding the additional mutational spectrum; the genes *APC*, *CDH1*, *CTNNB1*, *FGFR2*, and *STK11* were exclusively mutated in five individual early onset EACs. Whereas these mutations were not identified in the conventional EACs, that instead, exclusively carried additional mutations in the genes *ABL1*, *FBXW7*, *GNA11*, *GNAS*, *KRAS*, *MET*, *SMAD4*, and *VHL.* Since the additional mutations were identified in five individual early onset EACs it might be based on randomness. In addition, in a large whole exome sequencing study on EACs performed by *Dulak et al.* mutations in *APC*, *CDH1*, *CTNNB1*, *FGFR2*, and *STK11* were identified in EAC patients, here categorized as conventional EAC of older patients (range: 51-85 years), although in very small amounts [16].

However, considering the classification of cancer cell signaling pathways i.e. cell fate, cell survival, and genome maintenance, it is striking that the additional mutations in the early onset EACs occurred mainly in genes classified in cell fate pathways (*APC*, *CDH1*, *CTNNB1*), while all additional mutations in conventional EACs were identified in genes classified in survival pathways (*ABL1*, *GNA11*, *KRAS*, *MET*, *SMAD4*, *VHL, GNAS, FBXW7*). In addition, the shared mutations occurred in genes classified in genome maintenance pathways (*TP53*, *ATM*) as well as in survival pathways (*JAK3*, *PIK3CA*, *P16*). Mutations classified in cell fate pathways disturb the balance between differentiation and division, favoring the latter, which causes a selective growth advantage. Mutations categorized in cell survival pathways allow cancer cells to proliferate under limiting nutrient concentrations, making them survive in environments in which sister cells cannot [9]. Different biological pathways for patients with early onset cancers have been described earlier, for example in breast cancer, colorectal cancer, melanoma, and tongue cancer [18].

Taken together, these findings indicate that the development of EAC requires, regardless of the age of onset, a *TP53* mutation mostly accompanied by a *P16* mutation. However, the additional mutations needed to probably induce the malignant transformation [10] in some early onset EACs seem to occur in different genes, related to different pathways, as compared to the additional mutated genes in conventional EACs. From a treatment perspective, different pathways could indicate different inhibitors in the means of targeting treatment as has been established for metastatic colorectal cancer [19]. The current study gives a clue for a distinct molecular biology for early onset EAC. More extensive sequencing of larger cohorts of young adults and older patients with EAC have to be performed to determine whether early onset EAC is truly a distinct molecular entity that needs probably a different targeting therapy in the future.

## MATERIALS AND METHODS

### Patient identification

PALGA, the nationwide network and registry of histopathology and cytopathology, contains pathology reports generated in the Netherlands since 1971 and has complete national coverage since 1991 encompassing the pathology laboratories from all academic and nonacademic hospitals in the Netherlands [20]. The PALGA database was searched, with approval of their Privacy Commission and Scientific Council, to identify all patients diagnosed with an adenocarcinoma of the esophagus or the GEJ and aged ≤40 years in the Netherlands. The following search terms were used: “primary carcinoma,” “esophagus”, “stomach,” and “age ≤40 years”. The search was performed from January 1990 to March 2013. Cases were further confirmed or excluded after careful evaluation of the individual pathology reports.

The selected early onset EACs were compared with a group of conventional EACs collected from the Pathology archive of the Erasmus MC Cancer Institute, University Medical Center, Rotterdam. Patients aged ≥68 years and diagnosed with an adenocarcinoma of the esophagus or GEJ for which an esophagectomy was performed were selected and matched with the patients with early onset EAC with regard to grade of tumor differentiation and TNM-stage (according to the classification of the American Joint Committee on Cancer (AJCC) Staging Manual 7^th^ edition) [21].

### Tissue samples

Formalin fixed paraffin embedded (FFPE) tumor tissues were provided by the participating laboratories of PALGA. The tissue blocks were assessed anonymously according to the Proper Secondary Use of Human Tissue code established by the Dutch Federation of Medical Scientific Societies (http://www.federa.org). In addition the study was approved by The Medical Ethical Committee of the Erasmus MC Cancer institute, University Medical Center, Rotterdam.

Tumor tissue areas composed of at least 50% neoplastic cells (indicated by a GI-pathologist) were manually microdissected from 10 to 15 hematoxylin-stained sections (4μm) of FFPE tissue blocks. DNA was extracted using proteinase K and 5% Chelex 100 resin. To determine the presence of microsatellite instability analyses were performed with the MSI Analysis System, Version 1.2 (Promega, Madison, WI, USA).

### Next-generation sequencing

Ion semiconductor sequencing on the PGM was performed with the Ion AmpliSeq Cancer Hotspot Panel on tumor DNA according to the manufacturer's protocols. In short, libraries were made using the Ion AmpliSeq Library Preparation Kit. A template was prepared using the Ion OneTouch Template Kit and sequencing was performed with the Ion Sequencing Kit v2.0 on an Ion 316 chip. Data were analyzed with the Variant Caller v2.2.3-31149 (Life Technologies, Carlsbad, CA, USA). Variants were called when the position was covered at least 100 times. Variants found in at least 25% of the called reads were considered reliable. Variants present in the ESP6500si or 1000genomes databases in ≥1% were excluded. Subsequently nonsynonymous somatic point mutations, insertions and deletions that change the protein amino acid sequence and splice site alterations were selected as driver mutations.

### Data analysis

Early onset EAC was defined as patients diagnosed with EAC at the aged ≤40 years. This group was compared with the group of patients aged ≥68 years. Patient- and tumor characteristics were described using frequencies and percentages. Proportions were compared using χ2 test for categorical variables. Differences in the mean number of mutations between the age groups were tested using independent samples *T*-test. Two-sided *P*-values < 0.05 were considered statistically significant for all analyses. Data analysis was performed with SPSS version 20.0 (SPSS, Chicago, IL, USA).
